# Age and Time to Surgery Are Associated with Concomitant Meniscal Injuries in Adolescent ACL Tears: A Retrospective Cohort Study

**DOI:** 10.3390/healthcare14040491

**Published:** 2026-02-14

**Authors:** Marco Turati, Marco Caliandro, Edoardo Pierpaoli, Elena Tassistro, Stefania Galimberti, Antonio Andreacchio, Massimiliano Piatti, Giovanni Zatti, Marco Crippa, Marco Bigoni

**Affiliations:** 1 Orthopedic Department, Fondazione IRCCS San Gerardo Dei Tintori, 20900 Monza, Italy; 2 School of Medicine and Surgery, University of Milano-Bicocca, 20900 Monza, Italy; 3 Department of Orthopedic Surgery, Policlinico San Pietro, 24036 Ponte San Pietro, Italy; 4 Biostatistics and Clinical Epidemiology, Fondazione IRCCS San Gerardo Dei Tintori, 20900 Monza, Italy; 5 Bicocca Center of Bioinformatics, Biostatistics and Bioimaging (B4 Centre), School of Medicine and Surgery, University of Milano-Bicocca, 20900 Monza, Italy; 6 Pediatric Orthopedic Department, ‘Buzzi’ Children’s Hospital, 20154 Milan, Italy

**Keywords:** ACL tear, adolescent, concomitant injuries, meniscal tear, chondral injury

## Abstract

**Background**: Anterior Cruciate Ligament (ACL) tears are increasingly frequent among adolescents and are often accompanied by meniscal and chondral injuries that may compromise long-term outcomes. While risk factors are well documented in adults, they remain less well defined in younger patients, who present unique anatomical and activity-related characteristics. Understanding these associations is essential for optimizing surgical timing and outcomes in this age group. The purpose of this study was to determine the rate of concomitant intra-articular injuries in adolescents undergoing ACL reconstruction and to identify associated risk factors. **Methods**: We retrospectively reviewed 186 adolescents (12–18 years) who underwent primary ACL reconstruction using a bone–patellar tendon–bone (BPTB) autograft between 1999 and 2020. Demographic, anthropometric, and intraoperative findings on meniscal and chondral lesions were collected. Multivariable logistic regression models were used to identify factors associated with concomitant injuries. **Results**: Concomitant intra-articular lesions were found in 108 of 186 patients (58.1%). Meniscal tears were the most common finding, involving the lateral meniscus in 27.4%, the medial meniscus in 18.8%, and both menisci in 11.9%. The posterior horn was the most common tear location, and longitudinal and bucket handle patterns predominated. Older age was associated with any concomitant lesion (*p* = 0.028), and surgical delay (*p* = 0.021) was associated with a higher likelihood of medial meniscal tears. Sex and BMI were not significantly associated. **Conclusions**: Concomitant injuries are common in adolescents undergoing ACL reconstruction. Older age and delayed surgery were associated with a higher likelihood of meniscal damage, emphasizing the need for early diagnosis and timely surgical management as potentially relevant to limiting long-term degenerative changes in young athletes. **Level of evidence:** Level III, retrospective study.

## 1. Introduction

Anterior Cruciate Ligament (ACL) tears remain a major topic of debate in orthopedic and sports medicine, despite decades of research. While the adult population has been extensively investigated, considerably less attention has been devoted to children and adolescents [1].

In recent years, pediatric ACL tears have gained increasing attention due to a dramatic rise in incidence in this population. In the United States, ACL tears among individuals younger than 20 years old increased by 50% from 1994 to 2006 [2] and by an additional 19% from 2007 to 2011 [3]. The overall incidence of ACL tears in individuals aged 6 to 18 is estimated at 12.1 per 10,000 person-years, increasing by 2.3% annually until peaking at 17 years in boys and 16 years in girls [4]. In Europe, similar epidemiological trends have been reported. An Italian nationwide analysis showed a progressive rise in ACL reconstructions in patients younger than 15 years between 2001 and 2015 [5]. Northern European data also confirm an increasing incidence of pediatric ACL injuries, with studies from Finland and Norway reporting steady year-over-year growth in ACL tears and reconstructions among children and adolescents [6,7].

This trend is likely associated with the growing participation of children and adolescents in organized high-intensity sports activities [8] and underscores the growing clinical and social relevance of ACL injuries in the pediatric and adolescent populations.

Beyond isolated ligament disruption, ACL tears in adolescents are frequently accompanied by concomitant meniscal or chondral damage, which may compromise long-term outcomes. Werner et al. reported higher rates of associated meniscal and cartilage procedures in pediatric and adolescent patients undergoing ACL reconstruction compared with adults [3]. The presence of such lesions has been shown to accelerate degenerative joint changes and worsen postoperative function [9,10,11,12]. Notably, medial and lateral meniscal tears are not interchangeable in adolescents: in the ACL-deficient knee, the medial meniscus primarily contributes to limiting anterior tibial translation under anterior–posterior loads, while the lateral meniscus plays a greater role in restraining rotational and dynamic laxity [13].

While several studies in adults have identified surgical delay as the most important risk factor for secondary medial meniscal tears [14,15,16], evidence in younger patients remains limited and inconsistent. Conversely, female sex has been suggested as a protective factor [14,15,16,17,18], whereas the association with age and body weight remains unclear [15,17,18].

The relationship between these factors and the rate of concomitant injuries in children and adolescents has not yet been well defined, representing a critical gap in the literature. Defining these risk factors in adolescents is crucial, as timely diagnosis and management may help limit secondary joint damage in a population with long athletic careers ahead.

Therefore, the primary aim of this study was to describe the rate and characteristics of concomitant meniscal and cartilage injuries in a cohort of adolescent patients undergoing ACL reconstruction, to better understand incidence and severity of intra-articular damage.

The secondary aim was to evaluate the associations between concomitant lesions and potential risk factors, including time to surgery, age, sex, BMI and the type of sport played at the time of injury. We hypothesized that older age and a longer delay between injury and surgery would be significantly associated with a higher incidence of concomitant intra-articular lesions, particularly medial meniscal tears.

## 2. Materials and Methods

### 2.1. Study Design and Population

This retrospective cohort study was conducted in adolescents who underwent primary Anterior Cruciate Ligament (ACL) reconstruction between April 1999 and February 2020, before the onset of the COVID-19 pandemic, at a single high-volume orthopedic institution in Italy.

Patients were included in the study if they were adolescents (aged 12–18 years) with a diagnosis of a primary ACL tear who underwent ACL reconstruction using a bone–patellar tendon–bone (BPTB) autograft with transphyseal tunnels. The only exclusion criterion was a history of previous surgery on the ipsilateral knee (revision surgery), due to the risk of concomitant lesions from the previous injury. The diagnosis of an ACL tear was based on patient history, injury mechanism and a positive clinical examination, including the Lachman, anterior drawer and pivot–shift (jerk) tests, performed by an experienced orthopedic surgeon. Magnetic resonance imaging (MRI) was performed in all cases to confirm the clinical diagnosis and to assess concomitant injuries.

After diagnosis, patients were instructed to avoid physical exertion and refrain from twisting and pivoting activities until surgery. Timing of reconstruction depended on hospital scheduling and occasionally on the patient’s preference.

Institutional review board approval was obtained prior to study initiation.

### 2.2. Data Collection

Demographic, anthropometric, and clinical data were retrospectively retrieved from hospital records and imaging archives. For each patient, the following variables were collected: age at surgery, sex, body mass index (BMI), time from injury to surgery (days), and sport type at the time of injury. The index injury date was defined as the date of the ACL injury reported at the first orthopedic assessment (from the clinical history in the medical record). In case of documented re-injury, we referred to the index event leading to the confirmed ACL tear diagnosis and subsequent reconstruction, and we conservatively used the earliest clearly documented ACL injury date whenever multiple dates were reported. Surgical delay was calculated as the time interval between the index injury date and the date of ACL reconstruction. For descriptive purposes, surgical delay was also categorized using a 6-month threshold (≤180 vs. >180 days), selected as a pragmatic cutoff commonly used in clinical practice. Patient information was anonymized prior to analysis to preserve confidentiality.

Sports were categorized according to the degree of pivoting required using the three-level classification described by Hefti et al. [19]: Level I (high-pivoting), Level II (moderate), and Level III (low-pivoting), based on the sport at the time of the index injury as recorded in the clinical history. When sport information was not documented in the medical record, it was coded as missing and handled as described in Section 2.4.

Arthroscopic intra-articular findings were retrospectively extracted from operative reports completed at the time of surgery as part of routine clinical documentation by experienced orthopedic surgeons.

### 2.3. Surgical Technique

All procedures were performed by experienced orthopedic surgeons using a standardized technique. ACL reconstruction was performed using a BPTB autograft with transphyseal tunnels. During the initial arthroscopic phase of the procedure, a comprehensive evaluation of the entire joint was performed to identify any concomitant intra-articular lesions.

Meniscal tears were assessed both visually and with an arthroscopic probe. They were classified based on their location (anterior horn, body, posterior horn) and tear pattern (bucket handle, longitudinal, radial, peripheral, flap, double-flap, and complex). Management of meniscal tears was individualized according to tear type and tissue quality, with repair preferred whenever technically feasible in this young population.

Cartilage injuries were defined as focal chondral defects observed arthroscopically and graded according to the Outerbridge classification (grades I–IV) [20]. Except for one grade IV lesion, which was treated with autologous osteochondral transplantation, no surgical intervention was performed for cartilage injuries.

Postoperative rehabilitation followed an institutional protocol that remained largely consistent across the study period. Key elements included early restoration of range of motion, progressive strengthening and neuromuscular training, and criteria-based progression toward sport-specific activities and return to sport.

### 2.4. Statistical Analysis

The characteristics of the cohort, overall and stratified according to the presence or absence of concomitant lesions, were described as frequencies and percentages for categorical variables, and as mean (SD) or median (Q1–Q3) for continuous variables, as appropriate. Univariable analyses were conducted using the Chi-Square or Fisher’s exact test in case of categorical variables, and through the *t*-test or the Mann–Whitney in case of continuous variables.

Multivariable logistic regression models were used to assess the association of sex, age, BMI, sport at the time of injury (Level II–III vs. Level I), time from injury to surgery (in months, to facilitate effect interpretation) with the presence of any lesion, of lateral meniscal tears or of medial meniscal tears. Missing data were addressed using multiple imputation by chained equations, generating five imputed datasets. The imputation models included all covariates used in the regression analyses and the outcome variable. Imputation was performed over five iterations, and the results from each dataset were pooled according to Rubin’s rules. The performance of the models (pseudo R^2^ for goodness-of-fit, Brier Score for calibration and Area Under the Curve (AUC) for discrimination) were evaluated and presented as mean and standard deviation across imputed datasets. As a sensitivity analysis, a complete case analysis was performed to account for the presence of missing data. Linearity of the effect of continuous variables included in the logistic regression models was evaluated by fitting restricted cubic splines for continuous predictors and comparing model fit with linear specifications. Collinearity among covariates was assessed using variance inflation factors (VIFs).

Statistical analyses were performed with R version 4.4.1 (http://www.R-project.org, accessed on 10 October 2024). All tests were two-sided, with a significance level of 5%.

## 3. Results

### 3.1. Patient Demographics and Physical Characteristics

**Figure 1:**
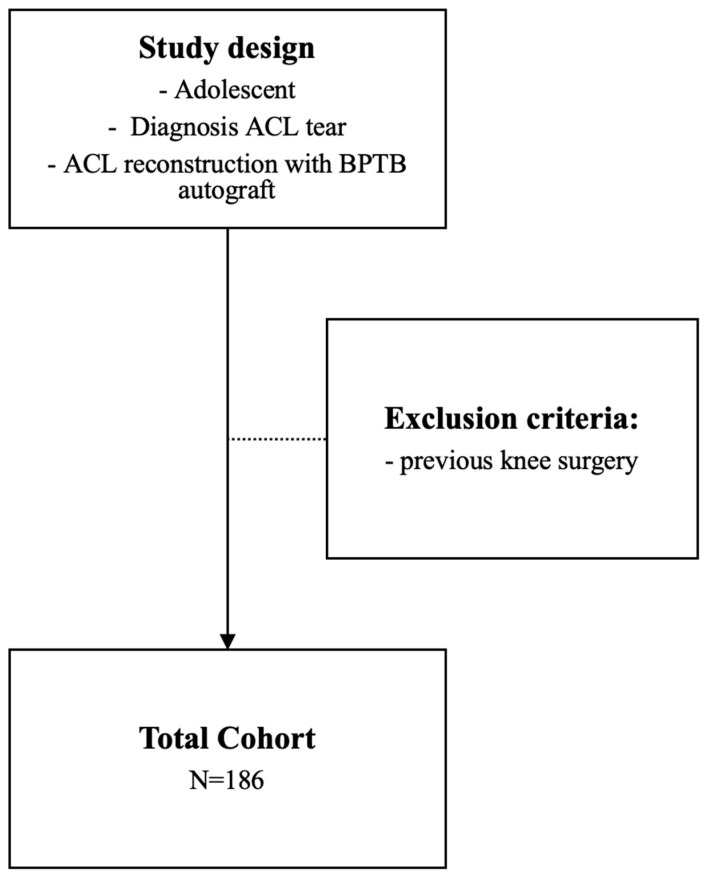
Flow chart with the number of patients.

A total of 186 adolescent patients were included in this study, comprising 128 males (68.8%) and 58 females (31.2%) (Table 1 and Figure 1).

The median age at the time of surgery was 16.4 (Q1–Q3 15.1–17.2) years, with 75 patients (40.3%) younger than 16 years. All patients had closing or closed physes at surgery, as confirmed by MRI [21].

The median time from injury to surgery was 149.9 (Q1–Q3 58.0–263.5) days. Ninety-seven patients (56.7%) underwent surgery within 180 days of the injury.

The median Body Mass Index (BMI) was 21.6 (Q1–Q3 20.2–23.6) kg/m^2^, with 154 patients (85.6%) having a BMI < 25 kg/m^2^.

Regarding the type of sport, 96 patients (70.1%) sustained the injury while playing high-pivoting sports (*Level I*), while only 4 patients (2.9%) were involved in low-pivoting activities (*Level III*).

### 3.2. Rate and Characteristics of Concomitant Lesions

Of the 186 patients, 78 (41.9%) had an isolated ACL tear, while 108 (58.1%) presented with at least one concomitant intra-articular lesion. Among these, 80 patients (74.1%) had a single lesion, 25 (23.1%) had two, and three (2.8%) presented with three or more associated injuries.

Demographic and physical characteristics of patients with and without concomitant lesions were largely comparable (Table 1). Age was the only variable that differed significantly between groups, whereas sex, BMI, sport type, and time to surgery were similarly distributed.

**Figure 2:**
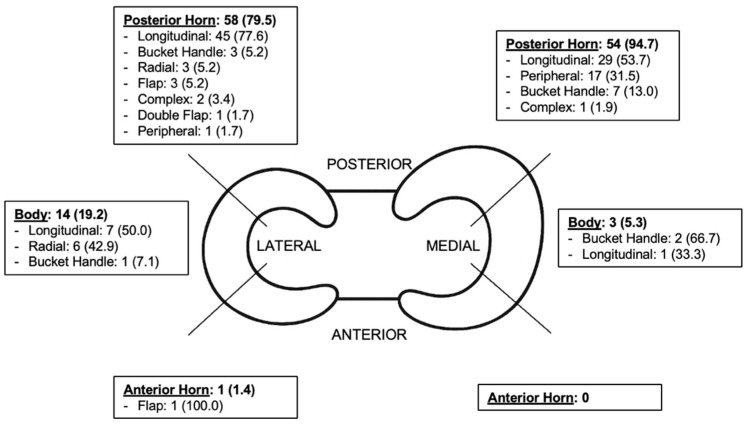
Distribution of meniscal tears by region and tear pattern for the medial and lateral menisci. Values are presented as N (%).

Meniscal tears were the most frequent associated injury. Lateral meniscal tears were found in 51 patients (27.4%), while medial meniscal tears were observed in 35 patients (18.8%). An additional 22 patients (11.9%) had tears involving both menisci (Figure 2 and Table 2).

The posterior horn was the most common tear location for both medial and lateral menisci.

Regarding the tear pattern, longitudinal tears predominated, representing 71.2% of lateral and 52.6% of medial lesions. Notably, bucket handle tears accounted for 15.8% of medial and 5.5% of lateral meniscal tears (Figure 2).

Chondral damage was less frequent, affecting the lateral tibial plateau (*n* = 4), the lateral femoral condyle (*n* = 3), and the medial femoral condyle (*n* = 3) (Table 3).

Only one patient had a concomitant posterior cruciate ligament (PCL) tear.

### 3.3. Potential Risk Factors

Multivariable logistic regression models after multiple imputation were performed to evaluate the association of potential risk factors with the presence of meniscal lesions (Table 4). The diagnostics and performance of the models were reported in the Supplementary Material (Table S1).

Older age (OR = 1.34, 95% CI = 1.03–1.73, *p*-value = 0.028; i.e., for each additional year of age, the odds of having any concomitant lesion increase by 34%) and participation in Level II–III sports (OR = 2.73 vs. Level I, 95%CI = 1.15–6.53, *p*-value = 0.027) were significantly associated with any concomitant lesion, whereas time from injury to surgery tended to be associated with the presence of a lesion (OR = 1.04, 95%CI = 1.00–1.08, *p*-value = 0.064) (Model A, Table 4).

No significant associations were found between sex, age, BMI, sport at injury time, time from injury to surgery and lateral meniscal or chondral lesions (Model B, Table 4).

A longer time from injury to surgery was associated with higher odds of medial meniscal tears (OR = 1.05, 95%CI = 1.01–1.09, *p*-value = 0.021, Model C, Table 4). As a sensitivity analysis, we also performed a complete case analysis (Supplementary Table S2) with consistent results.

## 4. Discussion

The present study analyzed the rate and characteristics of concomitant meniscal and chondral injuries in adolescents undergoing ACL reconstruction and identified potential risk factors associated with these lesions.

The main finding was the high rate of concomitant intra-articular injuries, with most patients showing damage to at least one additional structure. Older age at the time of surgery and a longer delay from injury to reconstruction were associated especially with medial meniscal tears.

Few studies have specifically analyzed concomitant ACL injuries in adolescents, and the reported rates vary widely, ranging from 23% to 43% for medial meniscus tears and 30% to 65% for lateral meniscus tears [22,23,24,25,26,27,28,29]. Our results align with a recent meta-analysis by Kay et al. [30], which reported a pooled prevalence of 33% for medial and 47% for lateral meniscal tears.

The posterior horn was the most frequently involved location in both menisci, consistent with prior studies by Henry et al. [28] and Samora et al. [26]. We also confirmed that longitudinal tears were the predominant pattern. Of particular interest was the high prevalence of bucket handle tears, reported in previous studies as the second most frequent pattern [23,26,28], with rates ranging from 9% to 29%. Our study corroborates this finding for the medial meniscus, where we observed a high rate of bucket handle tears. In contrast, for the lateral meniscus, we found that radial tears were the second most frequent pattern. This distinction is clinically relevant given the implications for the future knee health of young athletes.

Chondral lesions were less common in our cohort than in previous reports by Dumont et al. [22] and Kay et al. [30], who described rates of up to 31% for the medial femoral condyle and 19% for the lateral femoral condyle. This discrepancy may be explained by differences in classification methods, sample sizes, and surgical timing across studies.

This study investigated the relationship between potential risk factors and the presence of concomitant injuries.

In adult populations, surgical delay is a well-established risk factor for secondary medial meniscal tears [14,15,16]. While some authors [22,23,24,25,28,29,30,31] have reported a similar trend in the pediatric population, others have suggested a link between delayed surgery and both lateral meniscal [23,25,32] and chondral injuries [22,23,25,30,31]. Our findings are consistent with an association between longer time from injury to surgery and medial meniscal tears in adolescents. We hypothesize that this pattern may reflect prolonged functional instability and cumulative microtrauma during the waiting period. In addition, recent studies on the epiligament—the periligamentous connective tissue envelope containing microvasculature and reparative cells—suggest that limited intrinsic ACL healing capacity may contribute to persistent instability. This mechanistic interpretation remains speculative and warrants prospective confirmation [33,34].

Age at surgery was also associated with concomitant lesions, a trend consistent with the work of Dumont et al. [22] and Anderson et al. [23]. Interestingly, Anderson et al. [23] noted a decrease in lateral meniscal injuries with increasing age, whereas Dumont et al. [22] reported a corresponding increase in chondral damage. This may reflect progressive exposure to higher-impact sports and cumulative microtrauma with advancing skeletal maturity.

The influence of sport type remains controversial: our study found a tendency toward higher odds of concomitant lesions in patients participating in non-pivoting sports, while a similar analysis by Dumont et al. [22] found no clear association. Evidence from registry and cohort studies indicates that pivoting sports are associated with an increased incidence of complex concomitant injuries, while non-pivoting sports more frequently lead to isolated ACL tears [35,36]. Our finding should be interpreted cautiously: sport data were missing for 49 patients, and despite multiple imputation and consistent complete case sensitivity analyses, this association warrants confirmation in larger prospective cohorts.

Finally, BMI and sex were not associated with concomitant injuries in our results and their roles as risk factors remain debated. While Anderson et al. [23] reported female sex as a risk factor for medial meniscal injury, adult literature has often described it as a protective factor [14,15,16,17,18]. Similarly, while Vavken et al. [29] identified BMI as a risk factor, this is the only pediatric study to do so, with the exception of a study by Dumont et al. [22] that used body weight, a metric we consider less appropriate than BMI.

This study has several limitations that must be acknowledged. First, this is an observational study, and no conclusions about causality can be drawn from our findings also due to potential residual confounding. Second, its retrospective design relies on the quality and completeness of medical records, making it inherently susceptible to selection and information bias. A key example of this limitation is the missing sport data for 49 patients. We addressed this using multiple imputation and performed a complete case sensitivity analysis, which yielded consistent results; nevertheless, the amount of missingness and the small number of patients in some sport categories may limit precision, and these findings should be interpreted as exploratory. Although the overall sample size of our study is relatively modest, the number of events per variable included in the regression models is considered adequate for modeling purposes, ranging from a minimum of 11.4 events for each of the 5 covariates in the model to a maximum of 21.6, corresponding to the 57 medial lesions in the meniscal tears to the overall 108 lesions, respectively. Third, as a single-center study, our findings may have limited generalizability, as our patient population and surgical protocols may not be representative of other institutions. Additionally, the extended enrollment period (1999–2020) spans two decades of evolving surgical techniques, imaging quality, rehabilitation protocols, and diagnostic awareness, which may have introduced heterogeneity and may have led to underreporting of ramp and root tears, not routinely identified in the early years of the study. Finally, other potential risk factors (e.g., pivot–shift grading and posterior tibial slope) were not recorded in a systematic and reproducible manner across the study period; therefore, these potentially relevant factors could not be reliably retrieved for all patients and were not included in the analyses. These factors should be addressed in future studies with standardized documentation.

Therefore, future prospective studies with larger samples are necessary to more definitively determine the overall effect of these risk factors on concomitant injuries. Understanding these mechanisms could inform preventive strategies and optimize surgical timing in skeletally immature patients.

## 5. Conclusions

Concomitant injuries are highly prevalent among adolescents undergoing ACL reconstruction. Meniscal lesions—particularly posterior horn and bucket handle tears—represent the most common associated findings.

Older age and surgical delay were associated with a higher likelihood of concomitant meniscal injury in this cohort, underscoring the importance of early diagnosis and timely reconstruction as potentially relevant to preserving joint integrity and long-term outcomes in young athletes.

## Figures and Tables

**Table 1:** Demographic and physical characteristics of the enrolled population, overall and stratified by the absence or presence of concomitant injuries.

	Total (N = 186)	Absence of Lesions (N = 78, 41.9%)	Presence of Lesions (N = 108, 58.1%)	*p*-Value
Sex, N (%)				0.308
*Female*	58 (31.2)	28 (35.9)	30 (27.8)	
*Male*	128 (68.8)	50 (64.1)	78 (72.2)	
Age at surgery (years), median (Q1–Q3)	16.4 (15.1–17.2)	16.0 (14.9–17.0)	16.5 (15.4–17.3)	0.037
Age at surgery, N (%)				0.033
*<16* years	75 (40.3)	39 (50.0)	36 (33.3)	
*≥16* years	111 (59.7)	39 (50.0)	72 (66.7)	
BMI, median (Q1–Q3) (N = 180)	21.6 (20.2–23.6)	21.9 (20.5–23.6)	21.4 (20.1–23.5)	0.624
BMI, N (%) (N = 180)				0.999
*<25* kg/m^2^	154 (85.6)	66 (85.7)	88 (85.4)	
*≥25* kg/m^2^	26 (14.4)	11 (14.3)	15 (14.6)	
Sport at injury time, N (%) (N = 137)				0.447
*Level I*	96 (70.1)	43 (75.4)	53 (66.2)	
*Level II*	37 (27.0)	12 (21.1)	25 (31.2)	
*Level III*	4 (2.9)	2 (3.5)	2 (2.5)	
Time from injury to surgery (days), median (Q1–Q3) (N = 171)	149.9 (58.0–263.5)	141.0 (60.0–243.0)	162.5 (55.8–273.3)	0.430
Time from injury to surgery, N (%) (N = 171)				0.312
*<180* days	97 (56.7)	44 (62.0)	53 (53.0)	
*≥180* days	74 (43.3)	27 (38.0)	47 (47.0)	

**Table 2:** Demographic and physical characteristics of the enrolled population according to meniscal tear location.

	Lateral (N = 51, 27.4%)	Medial (N = 35, 18.8%)	Lateral and Medial (N = 22, 11.9%)	None (N = 78, 41.9%)
Sex, N (%)				
*Female*	15 (29.4)	9 (25.7)	6 (27.3)	28 (35.9)
*Male*	36 (70.6)	26 (74.3)	16 (72.7)	50 (64.1)
Age at surgery (years), median (Q1–Q3)	16.6 (15.5- 17.2)	16.4 (15.1–17.4)	16.4 (15.9–17.3)	16.0 (14.9–17.0)
Age at surgery, N (%)				
*<16* years	16 (31.4)	13 (37.1)	7 (31.8)	39 (50.0)
*≥16* years	35 (68.6)	22 (62.9)	15 (68.2)	39 (50.0)
BMI, median (Q1–Q3) (N = 180)	21.2 (20.2–23.5)	21.6 (19.5–23.2)	21.8 (20.2–24.1)	21.9 (20.5–23.6)
BMI, N (%) (N = 180)				
*<25* kg/m^2^	41 (83.7)	29 (87.9)	18 (85.7)	66 (85.7)
*≥25* kg/m^2^	8 (16.3)	4 (12.1)	3 (14.3)	11 (14.3)
Sport at injury time, N (%) (N = 137)				
*Level I*	25 (65.8)	15 (55.6)	13 (86.7)	43 (75.4)
*Level II*	11 (28.9)	12 (44.4)	2 (13.3)	12 (21.1)
*Level III*	2 (5.3)	0 (0.0)	0 (0.0)	2 (3.5)
Time from injury to surgery (days), median (Q1–Q3) (N = 171)	132.0 (40.8–231.8)	194.0 (127.0–368.0)	92.0 (41.0–299.0)	141.0 (60.0–243.0)
Time from injury to surgery, N (%) (N = 171)				
*<180* days	27 (58.7)	13 (39.4)	13 (61.9)	44 (62.0)
*≥180* days	19 (41.3)	20 (60.6)	8 (38.1)	27 (38.0)

**Table 3:** Distribution of cartilage damage severity in the lateral and medial compartments according to the Outerbridge classification.

	Total
Lateral Tibial Plateau, N (%) (N = 4)	
*Grade* *1*	2 (50.0)
*Grade* *2*	2 (50.0)
Lateral Femoral Condyle, N (%) (N = 3)	
*Grade 2*	1 (33.3)
*Grade 3*	1 (33.3)
*Grade 4*	1 (33.3)
Medial Femoral Condyle, N (%) (N = 3)	
*Grade* *1*	1 (33.3)
*Grade* *2*	2 (66.7)

**Table 4:** Results of the multivariable logistic regression models after multiple imputation for missing values on the presence of any lesion (Model A), of lateral meniscal lesions (Model B), of medial meniscal lesions (Model C).

Characteristic	Model A Presence of Any Lesion (Events = 108; N = 186)			Model B Presence of Lateral Lesions (Events = 73; N = 186)			Model C Presence of Medial Lesions (Events = 57; N = 186)		
	OR	(95% CI)	*p*-Value	OR	(95% CI)	*p*-Value	OR	(95% CI)	*p*-Value
Sex									
*Female*	Ref.	-	-	Ref.	-	-	Ref.	-	-
*Male*	1.79	(0.85–3.73)	0.125	1.08	(0.53–2.22)	0.933	1.73	(0.73–4.11)	0.216
Age at surgery (years)	1.34	(1.03–1.73)	0.028	1.22	(0.95–1.57)	0.116	1.18	(0.89–1.56)	0.247
BMI	0.95	(0.86–1.06)	0.360	1.01	(0.91–1.12)	0.856	0.97	(0.87–1.08)	0.543
Sport at injury time									
*Level I*	Ref.	-	-	Ref.	-	-	Ref.	-	-
*Level II–III*	2.73	(1.15–6.53)	0.027	0.96	(0.44–2.13)	0.927	2.37	(0.72–7.80)	0.182
Time from injury to surgery (months)	1.04	(1.00–1.08)	0.064	0.99	(0.97–1.02)	0.533	1.05	(1.01–1.09)	0.021

## Data Availability

The data presented in this study are available on request from the corresponding author. The data are not publicly available due to privacy restrictions.

## Supplementary Materials

The following supporting information can be downloaded at: https://www.mdpi.com/article/10.3390/healthcare14040491/s1, Table S1: Models diagnostics of the models performed after multiple imputation for missing values. Results are presented as mean (SD) across imputed datasets; Table S2: Results of the multivariable logistic regression models (complete-case analysis) on the presence of any lesion (Model A), of lateral meniscal lesions (Model B), of medial meniscal lesions (Model C).
