# Supplementary material for: Age and Time to Surgery Are Associated with Concomitant Meniscal Injuries in Adolescent ACL Tears: A Retrospective Cohort Study
Source: Healthcare (Basel). 2026 Feb 14;14(4):491. doi: 10.3390/healthcare14040491 (PMC12940482; doi:10.3390/healthcare14040491)
Supplement: Supplementary file 1 [file healthcare-14-00491-s001.zip › healthcare-4052447-supplementary.pdf]

Table S1. Models diagnostics of the models performed after multiple imputation for missing values. Results are presented as mean (SD) across imputed datasets.

**Index**	**Model A**<br>**Presence of any lesion**<br>**(N=186; events=108)**	**Model B**<br>**Presence of lateral lesions**<br>**(N=186; events=73)**	**Model C**<br>**Presence of medial lesions**<br>**(N=186; events=57)**
**Pseudo R^2**	0.106 (0.014)	0.026 (0.002)	0.110 (0.035)
**Brier Score**	0.225 (0.003)	0.234 (0.0003)	0.195 (0.006)
**AUC**	0.647 (0.017)	0.581 (0.003)	0.637 (0.037)

AUC = area under the curve

Table S2. Results of the multivariable logistic regression models (complete-case analysis) on the presence of any lesion (Model A), of lateral meniscal lesions (Model B), of medial meniscal lesions (Model C).

**Characteristic**	**Model A**<br>**Presence of any lesion**<br>**(N=130; events=76)**			**Model B**<br>**Presence of lateral lesions**<br>**(N=130; events=51)**			**Model C**<br>**Presence of medial lesions**<br>**(N=130; events=39)**		
	**OR**	**(95% CI)**	**p-value**	**OR**	**(95% CI)**	**p-value**	**OR**	**(95% CI)**	**p-value**
**Gender**									
Females	Ref.	-	-	Ref.	-	-	Ref.	-	-
Males	1.72	(0.69-4.45)	0.248	0.92	(0.38-2.26)	0.850	2.21	(0.80-6.71)	0.142
**Age at surgery (years)**	1.45	(1.09-1.96)	0.013	1.24	(0.93-1.67)	0.142	1.38	(1.02-1.93)	0.046
**BMI**	0.97	(0.85-1.10)	0.589	1.04	(0.91-1.18)	0.578	0.99	(0.86-1.13)	0.898
**Sport at injury time**									
Level I	Ref.	-	-	Ref.	-	-	Ref.	-	-
Level II-III	2.81	(1.08-7.93)	0.041	1.02	(0.40-2.55)	0.968	2.26	(0.81-6.57)	0.122
**Time from injury to surgery (months)**	1.02	(0.99-1.08)	0.326	0.97	(0.91-1.01)	0.192	1.05	(1.00-1.11)	0.076

OR=odds ratio; CI=confidence interval
